# Down Regulation of the TCR Complex CD3ζ-Chain on CD3+ T Cells: A Potential Mechanism for Helminth-Mediated Immune Modulation

**DOI:** 10.3389/fimmu.2015.00051

**Published:** 2015-02-18

**Authors:** Laura J. Appleby, Norman Nausch, Francesca Heard, Louise Erskine, Claire D. Bourke, Nicholas Midzi, Takafira Mduluza, Judith E. Allen, Francisca Mutapi

**Affiliations:** ^1^Centre for Immunity, Infection and Evolution, Institute of Immunology and Infection Research, School of Biological Sciences, University of Edinburgh, Edinburgh, UK; ^2^National Institutes of Health Research, Harare, Zimbabwe; ^3^Department of Biochemistry, University of Zimbabwe, Harare, Zimbabwe

**Keywords:** T cells, CD3ζ, schistosomiasis, human, helminth, downmodulation, antibody, pathology

## Abstract

The CD3ζ forms part of the T cell receptor (TCR) where it plays an important role in coupling antigen recognition to several intracellular signal-transduction pathways leading to T cell effector functions. Down regulation of CD3ζ leads to impairment of immune responses including reduced cell proliferation and cytokine production. In experimental models, helminth parasites have been shown to modulate immune responses directed against them and unrelated antigens, so called bystander antigens, but there is a lack of studies validating these observations in humans. This study investigated the relationship between expression levels of the TCR CD3ζ chain with lymphocyte cell proliferation during human infection with the helminth parasite, *Schistosoma haematobium*, which causes uro-genital schistosomiasis. Using flow cytometry, peripheral blood mononuclear cells (PBMCs) from individuals naturally exposed to *S. haematobium* in rural Zimbabwe were phenotyped, and expression levels of CD3ζ on T cells were related to intensity of infection. In this population, parasite infection intensity was inversely related to CD3ζ expression levels (*p* < 0.05), consistent with downregulation of CD3ζ expression during helminth infection. Furthermore, PBMC proliferation was positively related to expression levels of CD3ζ (*p* < 0.05) after allowing for confounding variables (host age, sex, and infection level). CD3ζ expression levels had a differing relationship between immune correlates of susceptibility and immunity, measured by antibody responses, indicating a complex relationship between immune activation status and immunity. The relationships between the CD3ζ chain of the TCR and schistosome infection, PBMC proliferation and schistosome-specific antibody responses have not previously been reported, and these results may indicate a mechanism for the impaired T cell proliferative responses observed during human schistosome infection.

## Introduction

The T cell receptor (TCR) complex, comprising of the TCR, a CD3ζ chain (CD3ζ) and CD3 co-receptor, has a tightly controlled assembly and expression within cells (1). CD3ζ is an integral part of the signaling pathway involved in TCR signaling (2), and its downregulation has been reported in numerous pathologies and conditions associated with chronic inflammation whilst the TCR on the cell surface remains present at normal concentrations (3). Thus, the CD3ζ chain is considered a “sensor” of sustained exposure to chronic inflammatory immune responses, a mechanism to restrict the magnitude of T cell responses and counteracting an overzealous immune reaction (3). Thus far there have been reports of a downregulated CD3ζ chain in pathologies such as cancers, arthritis, systemic lupus erythematosus, HIV, and leprosy, all conditions associated with impaired T cell functions (3–6).

The helminth parasite *Schistosoma haematobium* causes uro-genital schistosomiasis; a chronic condition typically associated with downregulated immune responses (7). Chronic inflammation associated with schistosomiasis is a hallmark of pathology (8, 9), and experimental models suggest that tissue inflammation is largely CD4+ T helper (Th) 2 driven (10, 11). It is unclear whether the TCR complex is modified in schistosomiasis in the same way as in the Th1 polarized inflammatory diseases in which CD3ζ has previously been studied (3). The immune response to schistosome infection typically consists of elevated levels of regulatory cytokines such as IL-10 and TGFβ (12, 13) and results in downregulation of T cell proliferation (14), cytokine production (15, 16), and hyporesponsiveness (17). Such a modulated immune response is characteristic of infected individuals in endemic environments who are believed to tolerate infection, facilitating parasite persistence, while at the same time limiting pathology associated with eggs laid by adult worms (18–20). The decreased proliferative capacity of peripheral blood mononuclear cells (PBMCs) during schistosome infection has been reported in both human and experimental studies (21–23), and serves to minimize pathology associated with host inflammatory responses (16). However, this downregulation and suppression of immune responses can also have spill-over effects into other areas of the immune response. For example, helminths are known to affect the host’s ability to mount an effective immune response following vaccination, leading to vaccine failure (24, 25). Mechanisms for downregulating the immune response in helminth infection have been investigated in experimental models, and focus primarily on myeloid cells and T regulatory cells (16, 26). Mechanisms associated with control and downregulation of the human immune response have generated interest from the fields of vaccine research, as well as of autoimmunity and allergy due to the potential for therapeutic interventions for these conditions (27–29).

We describe here levels of CD3ζ expression on T cells, PBMC proliferation, and antibody responses from a cohort of individuals living in a schistosome endemic area of rural Zimbabwe. We hypothesized that CD3ζ expression may be downregulated in chronic schistosomiasis and thus be related to *S. haematobium* infection levels within the cohort. Furthermore, we relate CD3ζ expression to schistosome-specific antibodies commonly associated with protection or susceptibility to infection. Our study is the first to show that CD3ζ expression on T cells is reduced during schistosome infection suggesting that this may be a mechanism for immune suppression in schistosomiasis.

## Materials and Methods

### Ethical approval

Ethical and institutional approval was granted by the Medical Research Council of Zimbabwe and the University of Zimbabwe’s Institutional Review Board. Local permission for the study was granted by the Provincial Medical Director. The study design, aims, and procedures were explained in the local language, Shona, prior to enrollment. Participants were free to drop out of the study at any time and informed written consent/assent was obtained from all participants and/or their guardians prior to taking part in the study and to receiving antihelminthic treatment.

### Study design

The study presented here was part of a larger on-going immuno-epidemiological study based in Mashonaland East, Zimbabwe where *S. haematobium* is endemic as is described elsewhere (30). The area has a low prevalence of soil transmitted helminths (STH) and *Schistosoma mansoni* (31), and the residents are subsistence farmers with frequent contact with *S. haematobium* infected water for purposes of bathing, washing, and collecting water. Recruitment into the study was school based and the wider community was also invited to participate. Residential history, antihelminthic treatment history, and water contact habits of the participants were captured through questionnaire. Following sample collection, participants were offered treatment with the antihelminthic drug praziquantel at the recommended dose of 40 mg/kg of body weight (32).

### Inclusion criteria

In order to be included in this study participants had to meet the following criteria: (1) be lifelong residents of the study area to allow age to be used as a proxy for history of exposure to schistosome infection, (2) have provided a minimum of two urine and two stool samples on consecutive days for parasite detection, (3) not have previously received antihelminthic treatment, (4) be negative for co-infection with malaria, STH, *S. mansoni*, and HIV, and (5) have provided a blood sample for serological and cellular assays. From an initial cohort of 633 recruited individuals, 68 were excluded for not meeting criteria 1–4 above and a further 184 did not provide sufficient blood sample for both serological assays and cell phenotyping. From the remaining 381 individuals, a cohort of 100 individuals was further selected to allow for, as far as possible, equal numbers of females to males and an even distribution of ages and infection prevalence. The final study group was dependent on the participant’s PBMC sample yielding at least 10^6^ cells to allow enough cells for all experimental conditions. The final study group consisted of 94 individuals and was divided into three age groups as described in Table 1.

**Table 1:** **Characteristics of study cohort**.

Infection status	Age group					
	5–10 years		11–15 years		>16 years	
	Sh−	Sh+	Sh−	Sh+	Sh−	Sh+
Sample size (no.)	25	15	17	13	15	9
Mean age in years (range)	7.76 (5–10)	7.60 (5–10)	13.06 (11–15)	12.54 (11–14)	29.73 (16–49)	27.11 (16–54)
Infection intensity	0	60.32	0	99.44	0	78.33
Infection range (SD)	0	1.33–185 (85.40)	0	0.33–523 (165.7)	0	0.33–550 (177.79)
Males:Females	8:17	11:4	9:8	8:5	2:13	3:6

*All selected people of the cohort were negative for HIV, soil transmitted helminths and *S. mansoni*; infection intensity: eggs/10 ml urine; Sh−, negative for *S. haematobium*, Sh+, positive for *S. haematobium**.

### Sample collection

From each participant, a stool and urine sample was collected on three consecutive days and examined microscopically for the presence of *S. haematobium* eggs in urine, and *S. mansoni* and STH eggs in stool using standard techniques (33, 34). Up to 20 ml of venous blood was collected from each participant in heparinized tubes and silicone-coated tubes (both from BD Biosciences, San Jose, CA, USA), for collecting PBMCs, and serum. An additional drop of blood was collected from each participant for microscopic detection of malaria parasites and for HIV detection using DoubleCheckGold™ HIV 1&2 Whole Blood Test (Orgenics Ltd., Yavne, Israel). PBMCs were isolated from the remaining tubes via density gradient centrifugation using Lymphoprep™(Axis-Shield, Cambridgeshire, UK). Isolated PBMCs were cryopreserved and stored in liquid nitrogen in Zimbabwe prior to freighting to Edinburgh in dry shippers.

### Antibody assays

Schistosome soluble worm antigen preparation (SWAP) specific antibody serum levels for IgA, IgE, and IgG4 were quantified using antibody ELISA. Lyophilized SWAP (Theodor Bilharz Institute, Giza, Egypt) was reconstituted as described by Mutapi et al. (35). ELISAs were conducted as previously reported (36), adding sample at a 1:20 dilution for IgA and IgE, and 1:100 dilution for IgG4 in 5% (weight/volume) skimmed milk powder. Secondary IgA HRP-conjugated antibody (A-7032 Sigma, St Louis, MO, USA) was added at a 1:1000 dilution, a 1:250 dilution for IgE (P-295, Dako, Glostrup, Denmark), and at a 1:500 dilution for IgG4 (MCA517, AbD Serotec, Oxford, UK). The colorimetric reaction was quantified with an ELISA reader at 405 nm. Each antibody ELISA was performed in duplicate on the same day for all samples with positive (high responders) and negative (Europeans who have never traveled to helminth endemic areas) controls included on all plates.

### Determining CD3ζ expression

Cryopreserved PBMCs were thawed as previously described (37), and resuspended at 5 × 10^6^ cells/ml in PBS. Cells were incubated with 10% FCS at 4°C for 10 min prior to staining for 30 min with PerCP-Cy5.5 conjugated anti-CD3 (clone OKT3 from eBiosciences, San Diego, CA, USA). Cells were permeabilized with permeabilization buffer [made up of 0.1% NaAzide and 0.1% saponin in Dulbecco’s PBS (Lonza, Verviers, Belgium)], and incubated with FITC conjugated anti-CD3ζ antibody (clone 6B10.2 from BioLegend, San Diego, CA, USA). At least 50,000 live events were acquired on an LSR II flow cytometer (BD Biosciences, San Jose, CA, USA). Analysis was performed using FlowJo software (TreeStar, USA) and mean fluorescence intensity (MFI) was calculated for CD3 and CD3ζ. CD3ζ expression on T cells was determined according to expression levels on CD3 positive cells, and normalized by subtracting the MFI of CD3 in the CD3 negative population.

### Proliferation assay

PBMCs were resuspended at 1 × 10^6^ cells per well, and stimulated with mitogens to induce proliferation. Cells were cultured for a total of 54 h at 37°C together with 50 ng/ml phorbol myristate acetate (PMA) and 1 μg/ml phytohemagglutinin (PHA) (Sigma-Aldrich, Dorset, UK), or cultured with X-VIVO medium as a negative control. After 36 h, the supernatant was removed and replaced with fresh X-VIVO medium containing tritiated thymidine (^3^H-Thymidine) (Amersham Biosciences, GE Healthcare, Little Chalfont, UK) at a final concentration of 0.1 μCurie/well. After a 18-h incubation at 37°C the plates were harvested and proliferation of the cell populations quantified according to ^3^H-Thymidine cellular incorporation. Uptake of ^3^H-Thymidine was quantified using a scintillation counter (Wallac-Perkin Elmer, MA, USA). Proliferation was quantified as counts per minute (cpm), and successful proliferation determined as >1000 cpm after media subtraction.

### Statistical analysis

All statistical analyses were conducted using the statistical package SPSS version 19 (IBM Corp., NY, USA). Data were analyzed using parametric linear regression. The data were transformed in order to meet assumptions of parametric tests. CD3ζ was measured as MFI of CD3ζ on CD3 positive cells after subtracting the CD3ζ MFI of the CD3 negative (non T cells) of the same individual. Final measurement of CD3ζ was square-root transformed. Antibody level (after subtraction of the blank control) was square-root transformed. Proliferation data, expressed as cpm were similarly square-root transformed after subtraction of media control values. Infection intensity was log transformed [log_10_ (*x* + 1)]. Categorical variables were sex (male/female), and age group [5–10 years (age group where infection is rising), 11–15 years (age group where infection is peaking), or >16 years (age group where infection is declining)].

Due to the possibility of gender and age dependent exposure patterns in this population (38, 39), it was necessary to allow for variation due to these factors prior to investigating the relationship of interest (40). The relationship between CD3ζ expression and infection intensity was determined via hierarchical linear regression analysis of infection intensity with CD3ζ expression, allowing for variation due to age and sex before testing for the relationship with infection intensity. The relationship between PBMC proliferation and CD3ζ expression for the whole cohort was determined using a linear regression analysis, allowing for variation due to sex, age group, and infection intensity. The relationship between CD3 and CD3ζ, as well as between SWAP IgA and SEA IgA, and IgE:IgG4 against SWAP was assessed for the whole cohort using a partial correlation analysis controlling for age group, infection intensity, and sex. For all statistical tests *p* ≤ 0.05 was considered significant.

## Results

### CD3ζ chain of the TCR is downregulated with increasing levels of infection

**Figure 1:**
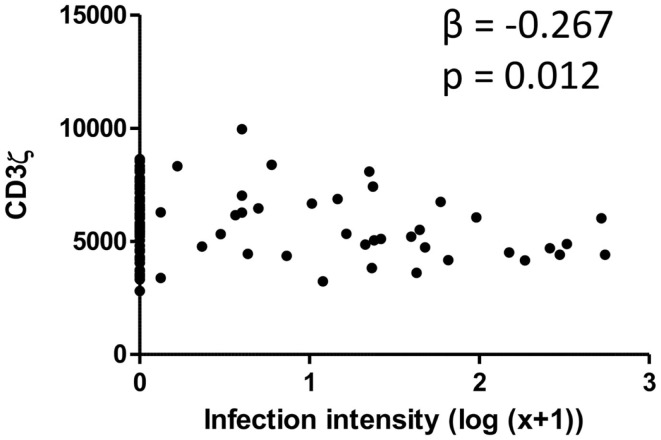
**Scatter graph of *Schistosoma haematobium* infection intensity [log_10_ (*x* + 1); *x* axis] and CD3ζ expression on T cells (MFI; *y* axis) for the whole cohort**. β and *p* values from linear regression analysis after allowing for age group (5–10, 11–15, 16+ years) and sex (male or female) are shown.

CD3ζ levels were measured on CD3+ T cells within the Zimbabwean cohort as an indication of activation status of the T cells. There was a significant and negative relationship between the intensity of *S. haematobium* infection and CD3ζ expression (Figure 1; Table 2). In order to confirm that this change was not related to an overall downregulation of the TCR complex (TCR) but due to a specific decrease in the CD3ζ chain, expression of the TCR co-receptor, CD3, was assessed in relation to CD3ζ expression as well as age and infection status. There was no significant relationship between CD3ζ and CD3 (*r* = −0.116, *p* = 0.282), confirming the relatively independent nature of CD3ζ expression within the TCR (3). Furthermore, the decrease in CD3ζ with infection intensity was not related to total levels of TCR expression, as CD3 levels were not significantly related to infection levels (β = 0.160, *p* = 0.124, see Figure S1 in Supplementary Material).

**Table 2:** **Coefficients of the regression model relating CD3ζ to schistosome infection intensity**.

	Standardized residuals		Unstandardized residuals		95% Confidence interval	
	β Value	*p* Value	Beta	SE	Lower bound	Upper bound
Constant			81.86	3.99	73.93	89.80
Sex	−0.08	0.46	−1.60	2.13	−5.82	2.63
Age group	−0.06	0.59	−0.69	1.27	−0.54	0.59
Infection intensity	−**0.27**	**0.01**	−**3.34**	**1.30**	−**5.93**	−**0.75**

*The table shows standardized and unstandardized coefficients from regression analysis. *R*^2^ = 0.073. Significant values are shown in bold. β value for infection intensity is represented on Figure 1*.

*SE, standard error*.

### CD3ζ levels are positively correlated with PBMC proliferation

**Figure 2:**
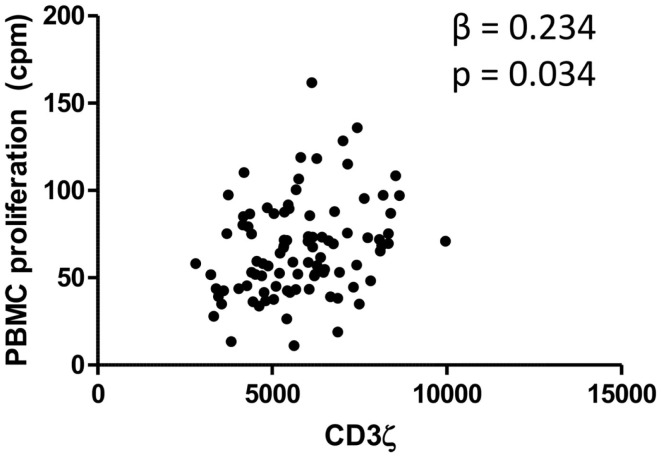
**Relationship between CD3ζ expression on T cells (MFI; *x* axis) and PBMC proliferation (cpm; *y* axis) for the whole cohort**. β and *p* values from regression analysis after allowing for age group (5–10, 11–15, 16+ years), sex (male or female), and infection intensity [log_10_ (*x* + 1)] are shown.

Levels of CD3ζ expression on T cells are intrinsically related to TCR activity and downstream immune responses, including cell proliferation (41). We thus related levels of CD3ζ expression on CD3 positive cells to the proliferative capacity of PBMCs from the whole cohort of schistosome exposed individuals. PBMCs from schistosome exposed individuals were stimulated for 54 h with PMA and PHA in order to assess proliferative capacity of the PBMCs independent of schistosome infection. The study showed a significant positive association between PBMC proliferation and CD3ζ expression. The relationship was significant after allowing for variation in sex, age, and infection intensity. Figure 2 shows the relationship between CD3ζ expression levels and PBMC proliferative capacity after stimulation in this population. CD3 expression was not related to PBMC proliferation (β = −0.025, *p* = 0.734).

Despite CD3ζ expression being significantly related to burden of infection, PBMC proliferation was not significantly related to the burden of schistosome infection (β = −0.168, *p* = 0.092).

### CD3ζ levels are negatively correlated to protective immune responses

Having shown the relationship between CD3ζ and infection intensity, we were interested in whether this was related to immune correlates to infection, as determined by antibody isotypes specific to adult schistosome antigens. Antibody production to the SWAP antigen was measured for IgA, IgG4, and IgE. Previous studies from this population show IgA to be a potential marker for susceptibility to infection (42–44). Relatively high levels of IgG4 are frequently produced in younger ages, thus it is considered to be a marker for susceptibility to infection. In contrast, IgE levels found to be higher in individuals who are more resistant to infection or reinfection (45, 46). Frequently, IgE and IgG4 are compared as ratios to better reflect changes in immunity on an individual level, such that high IgE:IgG4 is considered a marker for resistance to reinfection (45). As shown in Table 3, after controlling for sex and age group, IgG4 was found to be positively associated with infection intensity, while IgE and IgA did not show any significant relationship with infection intensity. IgE:IgG4 demonstrated a non-significant negative association with infection intensity.

**Table 3:** ***R* values from partial correlation between SWAP specific IgA, IgE, or IgG4 levels and IgE:IgG4 against infection intensity**.

	Infection intensity		
	*R* value	*p* Value	df
SWAP IgA	−0.013	0.901	86
SWAP IgE	0.117	0.277	86
**SWAP IgG4**	**0.280**	**0.009**	**83**
SWAP IgE:IgG4	−0.203	0.066	81

**R* values are from partial correlation, controlling for sex, and age group. Significant relationships are indicated in bold*.

*df, degrees of freedom*.

When relating levels of these antibodies to CD3ζ levels, IgA had a significant negative relationship with CD3ζ. There was also a significant negative correlation between IgE:IgG4 with CD3ζ. Table 4 shows the *r* and *p* values from the correlation analyses that were performed on the whole cohort.

**Table 4:** ***R* values from partial correlation between SWAP specific IgA, IgE, or IgG4 levels and IgE:IgG4 against CD3ζ expression**.

	CD3ζ		
	*R* value	*p* Value	df
**SWAP IgA**	−**0.292**	**0.006**	**85**
SWAP IgE	−0.182	0.092	85
SWAP IgG4	0.109	0.322	82
**SWAP IgE:IgG4**	−**0.254**	**0.022**	**80**

**R* values are from partial correlation, controlling for sex, age group, and infection intensity. Significant relationships are indicated in bold*.

*df, degrees of freedom*.

## Discussion

Both human and experimental studies have shown diminished cell proliferation in response to schistosome and bystander antigens during chronic schistosome infection (15, 22, 23, 47). These diminished responses are related to the presence of parasites (22, 48, 49). In limiting immune cell activation, pathology related to schistosome worm antigens is also limited (16, 50). The aim of this study was to investigate the relationship between expression levels of the TCR CD3ζ chain with lymphocyte cell proliferation during human infection with *S. haematobium* to determine if this is a possible mechanism through which T cell functions may be regulated.

CD3ζ levels are reported to be related to T cell responsiveness and proliferative capacity (2, 3, 41), and infection with schistosomes is associated with reduced proliferative responses (15, 16). Here, we investigated changes in the TCR CD3ζ chain in relation to schistosome infection intensity, and show a previously unreported negative association between CD3ζ levels and schistosome worm burden. In addition, we show a positive relationship between CD3ζ expression and PBMC proliferation, confirming the association between CD3ζ expression levels and PBMC proliferative capacity (3, 26). The observed downregulation of CD3ζ expression in conjunction with increasing infection intensity may be indicating a mechanism for downregulation of T cell proliferation in schistosomiasis. Downregulation of the immune response in schistosome infection is an important mechanism for modulating pathological host immune responses associated with parasite eggs; forming a balance between host immunity and successful parasite establishment (20). Indeed, in experimental models, where the ability to downregulate the immune response has been depleted, an influx of inflammatory cytokines results in increased pathological responses (12, 50).

PBMCs proliferation was not related to infection intensity. Both PHA and PMA directly stimulate or activate cells, bypassing the requirement for surface receptor stimulation (51, 52). The differing relationships between PHA/PMA stimulated PBMC proliferation and CD3ζ with infection intensity suggests that infection related downregulation of CD3ζ does not intrinsically impair cell function, and supports evidence of a reversible suppression of cell function related to the presence of schistosome worms (22).

Protective schistosome acquired immunity has been shown in several studies to be associated with high levels of IgE against adult worm antigens, and moreover a high IgE:IgG4 is associated with protection against reinfection (45, 46). In agreement with this, here we observed a negative association between IgE:IgG4 and infection intensity, as well as a positive and significant relationship between IgG4 and infection intensity, consistent with previous observations (53, 54).

In relating levels of CD3ζ to markers for specific schistosome immune responses, we show that IgA against SWAP was negatively related to CD3ζ levels, as was IgE:IgG4 against SWAP. The IgE:IgG4 is associated with developing immunity (45), indicating a potential relationship between developing immunity in conjunction with lower CD3ζ. IgG4 has been reported to be related to pathology (9, 55), thus the relationship of higher levels of CD3ζ with higher IgG4 levels in relation to IgE (low IgE:IgG4) supports the hypothesis that CD3ζ is downregulated as a protective mechanism against parasite related immune damage and may thus be associated with pathological immune responses. In contrast to IgE:IgG4, previous studies from Zimbabwe have shown IgA to be associated with susceptibility to infection, observing decreases in IgA levels with chemotherapeutic treatment (43), as well as lower levels in uninfected adults (42). Schistosomiasis is a disease, which displays dynamic changes in immune correlates throughout the course of infection (42, 56), with individuals living in endemic areas eventually developing protective immunity to infection (57, 58) and an immune profile that is skewed toward a Th2 dominant profile, while individuals who remain infected display a mixed Th1/Th2 environment (44). It is plausible that the downregulated T cell activity in schistosome infected individuals, observed here through decreased CD3ζ expression, may be contributing to the altered profiles seen in infection, not only preventing a pathological immune response, but also helping create an environment conducive to developing immunity toward infection. Further investigation into the relationship between CD3ζ and immune correlates are required to clarify the function of this marker during developing immunity.

Given that chronic schistosome infection can limit T cell responses to vaccine antigens (24, 27), and in previous studies in this cohort both auto-immune inflammation (59) and allergic reactivity (60) were reduced in people with higher schistosome infection, it is possible that the downregulation of the CD3ζ chain we observe may contribute to these phenomena. Indeed, the precise mechanisms leading to this downregulation of the CD3ζ chain in schistosome infection have important implications for fields such as vaccine development, where a fully functioning immune system is required to achieve optimal vaccine efficacy (61).

While we did not investigate the mechanisms leading to the downregulation of CD3ζ expression in schistosome infection, there may be an association with myeloid cells and myeloid derived suppressor cells (MDSC). For example, downregulation of the CD3ζ chain has previously been found to be related to an increase in activated MDSC in both human and murine studies (62, 63). Specifically in chronic schistosome infection, previous studies in this population, as well as elsewhere, have identified myeloid derived dendritic cells (mDCs) as having an altered expression level (37, 64). Furthermore, in experimental models of helminth infection, alternatively activated macrophages have been implicated as having a regulatory role (65). In humans, neutrophils have also been reported to act as modulators of T cell CD3ζ expression via l-arginine metabolism in inflammation and various pathological conditions (66, 67), and may be an area for further investigation in schistosome infection. Further identification of the mechanisms leading to immune suppression observed in helminth infection (22, 23), and the relationship between downregulation of CD3ζ observed here and clinical indices of helminth-mediated pathology is therefore warranted.

Overall, this study has shown a downregulation of CD3ζ levels in conjunction with increasing schistosome infection. Previous reports of CD3ζ expression being related to T cell activity, and the observation that levels of this marker are related to PBMC proliferation identifies downregulation of CD3ζ as a novel mechanism for immunoregulation during helminth infection in humans. Furthermore, we provide evidence of association between CD3ζ and markers for protective immunity. Mechanistic studies, perhaps including *in vitro* cell culture and blocking antibodies, will elucidate if the association is causal and also if elevated levels of CD3ζ expression mediate, or are a marker of, protective immunity.

## Author Contributions

Conceived and designed the study: FM, TM, and NM. Contributed to fieldwork: CB, NN, LA, TM, NM, and FM. Designed the experiments: JA, LA, NN, and FM. Performed the experiments: LA and FH. Analyzed the data: LA and FM. Wrote the paper: LA. Contributed to final version of the manuscript: LA, FM, CB, and NN.

## Conflict of Interest Statement

The authors declare that the research was conducted in the absence of any commercial or financial relationships that could be construed as a potential conflict of interest.

## Supplementary Material

The Supplementary Material for this article can be found online at http://www.frontiersin.org/Journal/10.3389/fimmu.2015.00051/abstract
