# Supplementary material for: Down Regulation of the TCR Complex CD3ζ-Chain on CD3+ T Cells: A Potential Mechanism for Helminth-Mediated Immune Modulation
Source: Front Immunol. 2015 Feb 18;6:51. doi: 10.3389/fimmu.2015.00051 (PMC4332365; doi:10.3389/fimmu.2015.00051)
Supplement: Supplementary file 1 [file Image_1.PDF]

Supplementary Figure:

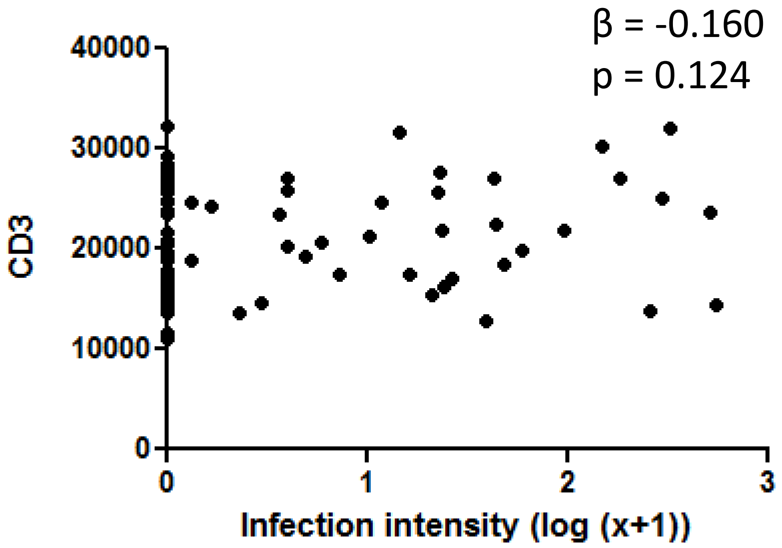

Figure shows scatter plot of infection intensity (x axis) and CD3 expression (y axis).
